# Supplementary material for: Using enriched semantic event chains to model human action prediction based on (minimal) spatial information
Source: PLoS One. 2020 Dec 28;15(12):e0243829. doi: 10.1371/journal.pone.0243829 (PMC7769489; doi:10.1371/journal.pone.0243829)
Supplement: Attachment — Submitted filename: Response_to_editor.pdf [file pone.0243829.s003.pdf]

First of all we would like to thank the editor for considering our work. In the following we are giving our response to the issue raised by the editor.

Editor Comment:

Despite your efforts, we found continued overlap between the submitted manuscript and the following publication, written by you/your-co-authors.

https://doi.org/10.1016/j.robot.2018.10.005

Please rephrase any duplicated text outside the methods section. The text that needs to be addressed involves lines:

104-107

115-121

131-144

Our response:

Thanks for your deep consideration. You are right. We did as you asked and changed the mentioned above lines in the main text.

Kind regards

Fatemeh Ziaeetabar, Ph.D.
